# Flow-mediated slowing shows poor repeatability compared with flow-mediated dilation in non-invasive assessment of brachial artery endothelial function

**DOI:** 10.1371/journal.pone.0267287

**Published:** 2022-05-24

**Authors:** João Luís Marôco, Marco Pinto, Helena Santa-Clara, Bo Fernhall, Xavier Melo

**Affiliations:** 1 Ginásio Clube Português, Research & Development Department, GCP Lab, Lisboa, Portugal; 2 Centro Interdisciplinar de Estudo da Performance Humana, Faculdade de Motricidade Humana, Universidade de Lisboa, Oeiras, Portugal; 3 College of Applied Health Sciences, University of Illinois at Chicago, Chicago, IL, United States of America; University of Massachusetts Boston, UNITED STATES

## Abstract

Pulse wave velocity (PWV) deceleration to reactive hyperemia–flow-mediated slowing (FMS)–has been suggested as an alternative method to flow-mediated dilation (FMD) to evaluate brachial artery endothelial function. FMS is suggested to address major caveats of the FMD procedure including its suboptimal repeatability and high-operator dependency. However, the repeatability of FMS has not been thoroughly examined, especially given the plethora of methods claiming to measure PWV. We assessed and compared the intra- and inter-day repeatability of FMS as measured by piezoelectric pressure mechanotransducers placed in the carotid and radial arteries, and brachial artery FMD as measured by echo-tracking. Twenty-four healthy male participants aged 23–75 yr, were examined on three separate days to assess intra and inter-day repeatability. All FMD and FMS examinations were conducted simultaneously by the same researcher complying with standardized guidelines. Repeatability was examined with intraclass correlation coefficient (ICC; >0.80), coefficient of variation (CV; <15%), and limits of agreement (95% LOA). Relative (%) FMD and FMS were scaled for baseline brachial artery diameter and PWV, respectively. Intra- (ICC: 0.72; CV: 136%; 95% LOA: -19.38 to 29.19%) and Inter-day (ICC: 0.69; CV: 145%, 95% LOA: -49.50 to 46.08%) repeatability of %FMS was poor, whereas %FMD demonstrated moderate-to-good intra- (ICC: 0.93; CV: 18%, 95% LOA: -3.02 to 3.75%) and inter-day repeatability (ICC: 0.74; CV: 25%, 95% LOA: -9.16 to 7.04%). Scaling FMD reduced the intra-day CV (-5%), and the uncertainty of the 95% LOA (- 37.64 to 35.69%) estimates of FMS. Carotid-radial artery FMS showed poorer repeatability compared to FMD.

## Introduction

Endothelial dysfunction is considered an early manifestation of atherosclerosis that precedes structural vascular alterations and consequently plays a key role in cardiovascular disease (CVD) pathogenesis [1, 2]. Endothelial dysfunction contributes to compromised tissue perfusion and exacerbates functional decline with age [3]. Thus, accurate and reproducible evaluation of endothelial function is of utmost importance not only for prognostic purposes [4] but also to quantify therapeutics and lifestyle interventions efficacy to ameliorate the risk of further development of atherosclerosis [5].

Brachial artery flow-mediated dilation (FMD) is a widely accepted non-invasive technique used to quantify endothelial function [6, 7]. The vasodilation imposed by reactive hyperemia after supra-systolic vascular occlusion represents the ability of peripheral conduit arteries to upregulate endothelial nitric oxide (NO) synthase in response to an increase in shear stress [8, 9]. Indeed, FMD is abolished with infusions of NO blockades (NG-monomethyl-L-arginine) [10, 11]. However, brachial artery FMD remains to be recommended for routine clinical use [12] owing to both within and between participants variability and being technically challenging, requiring experienced and well trained operators [9, 13], but also the lack of real consensus regarding methodological confounders, analysis, and interpretation of results [9]. One of those methodological confounders is resting brachial artery diameter (D_bas_), which is negatively associated with FMD [14] and could explain ~65% of its variability. This could lead to biased estimates of FMD if not allometrically corrected [15], but whether this approach improves repeatability remains unknown.

Alternatively, flow-mediated slowing (FMS) has been suggested as an apparently simpler and more reproducible [16] method to indirectly assess endothelial function, while addressing some of the caveats credited to FMD, including its suboptimal repeatability and high-operator dependency. This method measures the deceleration of brachial pulse wave velocity (PWV)—a surrogate marker of conduit artery stiffness–in response to reactive hyperemia stimulus [17]. According to the Moens–Korteweg equation [18], the deceleration of PWV is thought to result from an increase in NO-mediated arterial dilation [19, 20].

FMS could be useful as an indicator of vasomotor function suitable for evaluation of large-scale populations and early-stage disease cohorts but to date, the repeatability of FMS has not been thoroughly evaluated. The only study to date aimed to examine the repeatability of FMS [16] measured PWV using the brachial-radial oscillometric technique (Vicorder, Skidmore medical, Bristol, UK). This is particularly relevant as a plethora of non-invasive devices are commercially available, incorporating different techniques to measure PWV. These devices are not interchangeable in their use, as the elastic properties of the arterial segments incorporated in the analysis (carotid, brachial, radial arteries) assessed differ between devices and are likely to yield different estimates of PWV to reactive hyperemia [21]. For example, the piezoelectric pressure mechanotransducers technique (Complior, Alam Medical, France) estimates regional PWV over both elastic (i.e., carotid and aorta) and muscular (i.e., brachial and radial) upper limb arteries, whilst the more local PWV estimates from the brachial-radial oscillometric technique only use muscular arteries. Thus, the repeatability of PWV estimates derived from piezoelectric mechanotransducers might not be interchangeable with the repeatability of PWV estimates derived from the oscillometric technique. Therefore, this study aimed to 1) assess and compare the intra- and inter-day repeatability of FMS as measured by piezoelectric pressure mechanotransducers placed in the carotid and radial arteries, and brachial artery FMD as measured by echo-tracking 2) and to examine the impact of allometrically scaling FMD and FMS to D_bas_ and baseline carotid-radial PWV, respectively, on the repeatability of these measurements in apparently healthy young and older male adults.

## Methods

### Participants

Twenty-four healthy and physically active male participants aged 23–75 yr were recruited to participate in the study. Only healthy participants were recruited as FMD [9] and assumingly FMS, are less repeatable in populations with cardiovascular risk factors and chronic diseases. Participants were recruited both from the professional network of Ginásio Clube Portugês and the Faculdade de Motricidade Humana, by email and face-to-face conversations. Exclusion criteria were as follows: any sources of nicotine use, body mass index > 30 kg/m^2^, brachial systolic and diastolic blood pressures > 140/90 mmHg, cardiovascular (e.g., heart failure, coronaropathy), metabolic (e.g., diabetes mellitus), and renal diseases. All participants were physically active (M = 200, SD = 25 min/wk) according to the results of the International Physical Activity Questionnaire (IPAQ). All participants reported to the laboratory on a fasted state (≥ 6h) and refraining from strenuous exercise, foods/drinks containing caffeine and alcohol, and vitamin C rich foods and supplements (≥ 12h). This latter requirement is important because it has been suggested that vitamin C restores or even upregulates FMD through its anti-oxidant effects in older adults with endothelial dysfunction [22]. Inter-day repeatability was assessed on two occasions at the same time of the day (in the morning) with a minimum of 48h between sessions. Intra-day repeatability was assessed with two measurements performed 20 min apart, with the second measurement performed only after brachial artery diameters returned to baseline values. Both FMD and FMS measurements were performed simultaneously. All participants gave written informed consent after a detailed explanation of the experimental procedures and aims of the study. All experimental procedures were approved by the ethics committee of Faculdade de Motricidade Humana–Universidade de Lisboa (10/2020) and were aligned with the Declaration of Helsinki for human research.

#### Flow-mediated dilation

FMD was assessed in the right brachial artery with an ultrasound (Arietta V60, Hitachi Aloka Medical Ltd, Mitaka-shi, Tokyo, Japan) equipped with a 7.5-MHz linear array probe incorporating a 5-MHz Doppler transducer, placed ~4 cm above the antecubital fossa, and held by a mechanical clamp following standard guidelines [8, 9]. Before each measurement, participants were in a supine position for 15 min and had their brachial systolic and diastolic blood pressure measured using an automated digital device (HEM-7361T-EBK) manufactured by Omron Healthcare (Kyoto, Japan) with their right arms extended <80° laterally from the torso and at the level of the heart, in a quiet climate control room (22-24°C). To ensure that the same brachial arterial region was imaged, pictures of the setup were taken, and color markers were placed nearby the probe for inter and intra-day measures, respectively. Reactive hyperemia was induced by rapid cuff-deflation following a forearm occlusion (Hokanson SC10, Bellevue, WA 98005, USA) maintained for 5 min at 250 mmHg. Intraluminal brachial artery diameter was measured with automated edge-detection software allowing precise measurement of the artery diameter [9]. Off-line analyses of FMD were conducted on the in-built software provided by the manufacturer (SOP-ARIETTA60-16, Hitachi Aloka Medical Ltd, Mitaka-shi, Tokyo, Japan). D_bas_ was averaged in end-diastole during the last 60-s of the baseline period, whereas the highest 10-s average interval throughout the first 3-minute collection period after cuff-deflation represented peak hyperemic diameter (D_peak_). FMD was calculated both as an absolute change ($FMD\;[mm]={peak\;diameter\;}_{post-ischemia\;}-D_{bas}$) and as a relative change ($\%FMD=\frac{D_{peak}-D_{bas}}{D_{bas}}\times 100\%$) in diameter. %FMD was also allometrically scaled for D_bas_ (scaled FMD) in each measure of intra and inter-day analyses as a non-linear ratio between D_peak_ and D_bas_ was observed (allometric β unstandardized coefficients < 1) [15]. Thus, D_bas_ was introduced in the linear regressions as the independent variable and %FMD as the dependent variable, and the unstandardized individual residuals were added to the mean %FMD of each participant [23]. All image acquisitions and analyses were performed by the same researcher who had more than 100 hours of experience.

#### Flow-mediated slowing

Baseline and post-occlusion induced ischemia PWV of the right carotid-radial tract (crPWV) was measured using a non-invasive automatic device (Complior, Alam Medical, France). Participants were in a supine position on a cushioned table with their right arm positioned 70-80° to their body during the PWV measurement [16] The common carotid artery and radial artery pressure waveforms were recorded using 2 piezoelectric pressure mechanotransducers placed on both arteries. The travel time distance(d) was obtained before each measurement by the same operator and was defined as recommended by the user’s manual as the tape measured distance over the suprasternal notch to the styloid process of the radius with the arm at 90° from the torso interest [21]. The pulse transit time (PTT) was automatically calculated, allowing the calculation of PWV as $PWV=\frac{d}{PTT}$. Measures of 10 consecutive pulse waveforms were duplicated until a difference of <0.5 m/s in crPWV was obtained [24] at the last min of the baseline period and at min 1, 2, and 3 following a 5-minute cuff occlusion (250 mmHg). crPWV measurements with the highest quality index (> 90%), calculated as $Quality\;index=100-100\times \frac{SD\;of\;TT}{Average\;of\;TT}$, were used for the final analysis. Similarly to FMD, FMS was calculated both as an absolute-change ($FMS\;[m/s]=crP{PWV}_{post-ischemia\;}-{crPWV}_{bas}$) and a relative change between baseline PWV and post-deflation PWV ($\%FMS=\frac{cr{PWV\;}_{min\;}–cr{PWV\;}_{bas}\;}{cr{PWV\;}_{bas}}\times 100\%$) [16]. %FMS was also allometrically scaled for baseline crPWV (scaled FMS) for each measurement, following similar procedures as described for FMD, because a non-linear ratio between crPWV_post-ischemia_ (dependent variable) and crPWV_bas_ (independent variable) was observed (β < 1). Noisy signals were observed in intra- (3 participants) and inter-day (1 participant) measurements of FMS, which made the acquisition of 10 high-quality pulse waveforms not feasible.

### Anthropometrics, body composition

Body composition parameters (free fat mass and fat mass) were assessed with a bioimpedance device (seca mBCA 515, seca gmbh & co. kg, Hamburg, Germany) that uses four pairs of electrodes positioned at each hand and foot. Height and body weight were measured to the nearest 0.1 cm and 0.1 kg on a scale with an attached stadiometer (model 770, Seca, Hamburg, Deutschland).

### Statistics analysis

Based upon an ICC estimate of 0.80 from the FMD repeatability results of high specialized vascular laboratories (operators with complete certification process where 10 repeat scans with a coefficient of variation <2% %FMD are required) [25], an à priori power analysis using R package ICC.Sample.Size [26] suggested that 24 participants were necessary to ensure good repeatability in intra and inter-day repeated measurements (α = 0.05, 1−β = 0.90, k = 2, null hypothesis = 0.40).

The distributions of FMD and FMS were checked for normality with the Shapiro-Wilk test and plot representation. FMS was not normally distributed but the difference between repeated measurements was. Repeatability assessments of FMD and FMS were conducted using absolute (coefficient of variation (CV) calculated as (SD/Mean) * 100)) and relative measures (non-parametric intraclass correlation coefficient [27]) computed with the nopaco package [28]. The ICC was interpreted as: poor < 0.50, moderate [0.50, 0.74], good [0.75, 0.90], and excellent > 0.90. [29], whereas CV < 15% were indicative of good repeatability [9]. Bland-Altman plots were also used to assess the repeatability of both methods, using ggplot 2 package [30]. The association between FMD and FMS was tested with repeated measures correlation coefficient using the rmcorr package [31]. All statistical analyses were conducted using R version 4.0.3 [32] with a significant level set at (α) < 0.05.

## Results

### Characteristics of the participants

The clinical and demographic characteristics of the participants are depicted in Table 1.

**Table 1:** Characteristics of the participants (n = 24).

Characteristic	
Age (years)	45 (19)
Height (m)	1.75 (0.06)
Weight (kg)	78.47 (9.32)
Body mass index (kg/m^2^)	25.8 (2.4)
Waist circumference (m)	0.91 (0.09)
Fat mass (kg)	21.3 (6.5)
Free fat mass (%)	61.5 (6.7)
bSBP (mmHg)	125 (12)
bDBP (mmHg)	75 (9)
HR (b.min^-1^)	60 (8)
FMD (%)	6.01 (3.43)
FMS (%)	-1.23 (10.25)
D_bas_ (mm)	4.00 (0.60)
crPWV_bas_ (m/s)	9.45 (1.11)

Data presented as mean (SD). Abbreviations: bSBP: brachial systolic blood pressure; bDBP: brachial diastolic blood pressure; FMD: flow-mediated dilation; Dbas: resting brachial artery diameter; FMS: flow-mediated slowing; crPWVbas: carotid-radial pulse wave velocity.

### Repeatability of FMS and FMD

The intra-day descriptive statistics for FMS and FMD and other selected parameters are detailed in S1 File. There was large variability between participants in the main outcomes as portrayed by the large standard deviation (SD) and CV.

Intra-day repeated measurements of %FMS showed overall poor repeatability, even if ICCs of intra-day measurements of FMS showed a moderate correlation. In contrast, FMD showed good repeatability supported by the ICC, and the respective narrow confidence interval (Table 2). Scaling FMS to crPWV failed to improve the reproducibility of FMS when compared to the %FMS measurement (Table 2), whereas scaling FMD to D_bas_ reduced the CV by 5%. Absolute FMS (m/s) and FMD (mm) repeated measurements did not show better repeatability compared to %FMS, % FMD, scaled FMS and %FMD, respectively, as supported by the ICCs in Table 2. All parameters used to calculate FMS (e.g., crPWV_bas_) and FMD (D_bas_ and D_peak_) exhibited CVs < 10%.

**Table 2:** Intra-day repeatability statistics.

			95% ICC Confidence Interval	
Variables	CV (%)	ICC	Lower bound	Upper bound
FMD (%)	18	0.89	0.83	1
Absolute FMD (mm)	20	0.90	0.83	1
Scaled FMD (%)	13	0.91	0.84	1
FMS_i_ (%)	123	0.74	0.65	1
Absolute FMS_i_ (m/s)	115	0.73	0.65	1
FMS_ii_ (%)	126	0.74	0.63	1
Absolute FMS_ii_ (m/s)	137	0.72	0.63	1
FMS_iii_ (%)	136	0.72	0.63	1
Absolute FMS_iii (_m/s)	134	0.75	0.66	1
Scaled FMS_i_ (%)	113	0.79	0.71	1
Scaled FMS_ii_ (%)	129	0.79	0.70	1
Scaled FMS_iii_ (%)	180	0.74	0.64	1
D_bas_ (mm)	3	0.95	0.90	1
D_peak_ (mm)	3	0.96	0.91	1
crPWV_bas_ (m/s)	7	0.83	0.76	1
crPWV_i_ (m/s)	10	0.81	0.74	1
crPWV_ii_ (m/s)	10	0.80	0.73	1
crPWV _iii_ (m/s)	8	0.79	0.72	1

Non-parametric intraclass correlation coefficients (ICC) and coefficients of variation (CV) were calculated over two measurements. Abbreviations: FMD: flow-mediated dilation, FMS: flow-mediated slowing at 1^st^ (FMS_i_), 2^nd^ (FMS_ii_), and 3^rd^ (FMS_iii_) minute post-occlusion; Dbas: brachial artery resting diameter; Dpeak: reactive hyperemia peak brachial artery diameter; crPWVbas: baseline carotid-radial pulse wave velocity; crPWV: carotid-radial pulse wave velocity at 1^st^ (crPWV_i_), 2^nd^ (crPWV_ii_) and 3^rd^ (crPWV_iii_) minute post-occlusion. FMS data were missing for three participants.

**Fig 1:**
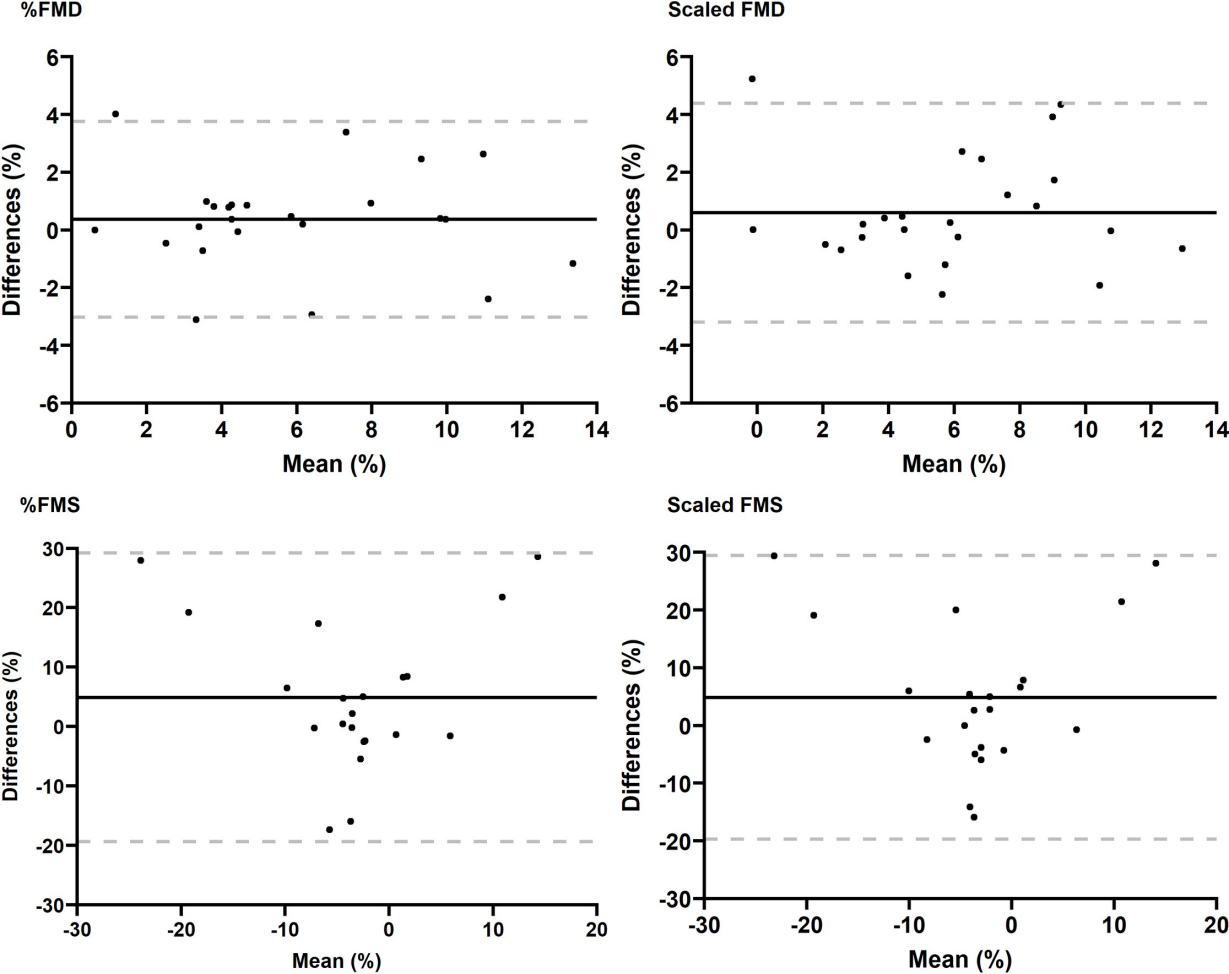
Bland-Altman plots for intra-day FMD and FMS measurements. Y-axis represents the difference between the first and second measures; the dashed grey lines correspond to the 95% LOA and the black line represents the mean of the differences. For %FMD, the lower LOA was– 3.02% (95% CI: - 4.29 to– 1.76); and the upper LOA was 3.75% (CI: 2.48 to 5.01); for scaled FMD, the lower LOA was—3.19% (95% CI: - 4.614 to -1.78); and the upper LOA was 3.99% (95% CI: 2.57 to 5.40); for %FMS, the lower LOA was—19.38% (95% CI: -28.45 to -10.32) and the upper LOA was 29.19% (CI of 20.13 to 38.26); for the scaled FMS, the lower LOA was -18.92% (95% CI: - 27.13 to– 10.71); and the upper LOA was 29.67% (95% CI: 21.45 to 37.87). FMS data were missing in 3 participants.

The Bland-Altman plots for intra-day FMS showed poor repeatability, especially those of the 1^st^ and 2^nd^ min post occlusion (S2 File). With respect to the 3^rd^ min post occlusion, the bias between measurements was 4.86% (SD = 12.54) and 5.37% (SD = 11.23) for %FMS and scaled FMS, respectively. Conversely, Bland-Altman plots for intra-day FMD measurements showed smaller bias for both unscaled (0.36% (SD = 1.73)) and scaled (0.60% (SD = 1.93)) FMD. Three participants had missing data concerning intra-day measurements of FMS and were excluded from the final analysis of intra-day reproducibility. Bland-Altman plots for intra-day FMD showed no evidence of proportional bias and only one participant did not fall within the 95% limits of agreement (LOA) (Fig 1).

The inter-day descriptive statistics are detailed in S4 File and the inter-repeatability statistics for FMS and FMD, as well as other selected parameters, are detailed in Table 3, respectively.

**Table 3:** Inter-day repeatability statistics.

			95% ICC Confidence Interval	
Variables	CV (%)	ICC	Lower bound	Upper bound
FMD (%)	25	0.78	0.70	1
Absolute FMD (mm)	25	0.78	0.69	1
Scaled FMD (%)	31	0.74	0.66	1
FMS_i_ (%)	119	0.72	0.64	1
Absolute FMS_i_ (m/s)	143	0.72	0.64	1
FMS_ii_ (%)	133	0.68	0.61	1
Absolute FMS_ii_ (m/s)	133	0.68	0.60	1
FMS_iii_ (%)	145	0.69	0.61	1
Absolute FMS_iii_ (m/s)	94	0.75	0.66	1
Scaled FMS_i_ (%)	153	0.71	0.64	1
Scaled FMS_ii_ (%)	233	0.67	0.59	1
Scaled FMS_iii_ (%)	145	0.70	0.62	1
D_bas_ (mm)	3	0.90	0.84	1
D_peak_ (mm)	3	0.96	0.91	1
crPWV_bas_ (m/s)	8	0.77	0.68	1
crPWV_i_ (m/s)	12	0.71	0.64	1
crPWV_ii_ (m/s)	12	0.33	0.68	1
crPWV _iii_ (m/s)	11	0.75	0.66	1

Intraclass correlation coefficients (ICC) and coefficients of variation (CV) were calculated over two measurements. Abbreviations: FMD: flow-mediated dilation, FMS: flow-mediated slowing at 1^st^ (FMS_i_), 2^nd^ (FMS_ii_), and 3^rd^ (FMS_iii_) minute post-occlusion; D_bas_: brachial artery resting diameter; D_peak_: reactive hyperemia peak brachial artery diameter; crPWV_bas_: baseline carotid-radial pulse wave velocity; crPWV: carotid-radial pulse wave velocity at 1^st^ (crPWV_i_), 2^nd^ (crPWV_ii_) and 3^rd^ (crPWV_iii_) minute post-occlusion. FMS data were missing for one participant.

The inter-day repeatability measurements of FMS were similarly poor as suggested by the large CV, even if inter-day ICCs for FMS were moderate. Inter-day measurements of FMD showed lower repeatability compared to intra-day measurements of FMD as supported by the larger CVs (Table 2). Inter-day repeatability measurements of scaled FMS and FMD were identical to %FMS, FMS (m/s), %FMD and FMD (mm), respectively, as supported by the ICCs in Table 3. A supplemental age group analysis is available in S3 File. Overall, older adults showed better inter-day repeatability measurements of FMD compared to young adults but not in intra-day measurements. Moreover, no significant association was found between age and %FMD (r (22) = 0.07, *p* = 0.76) or %FMS (r (20) = 0.19, *p* = 0.40).

**Fig 2:**
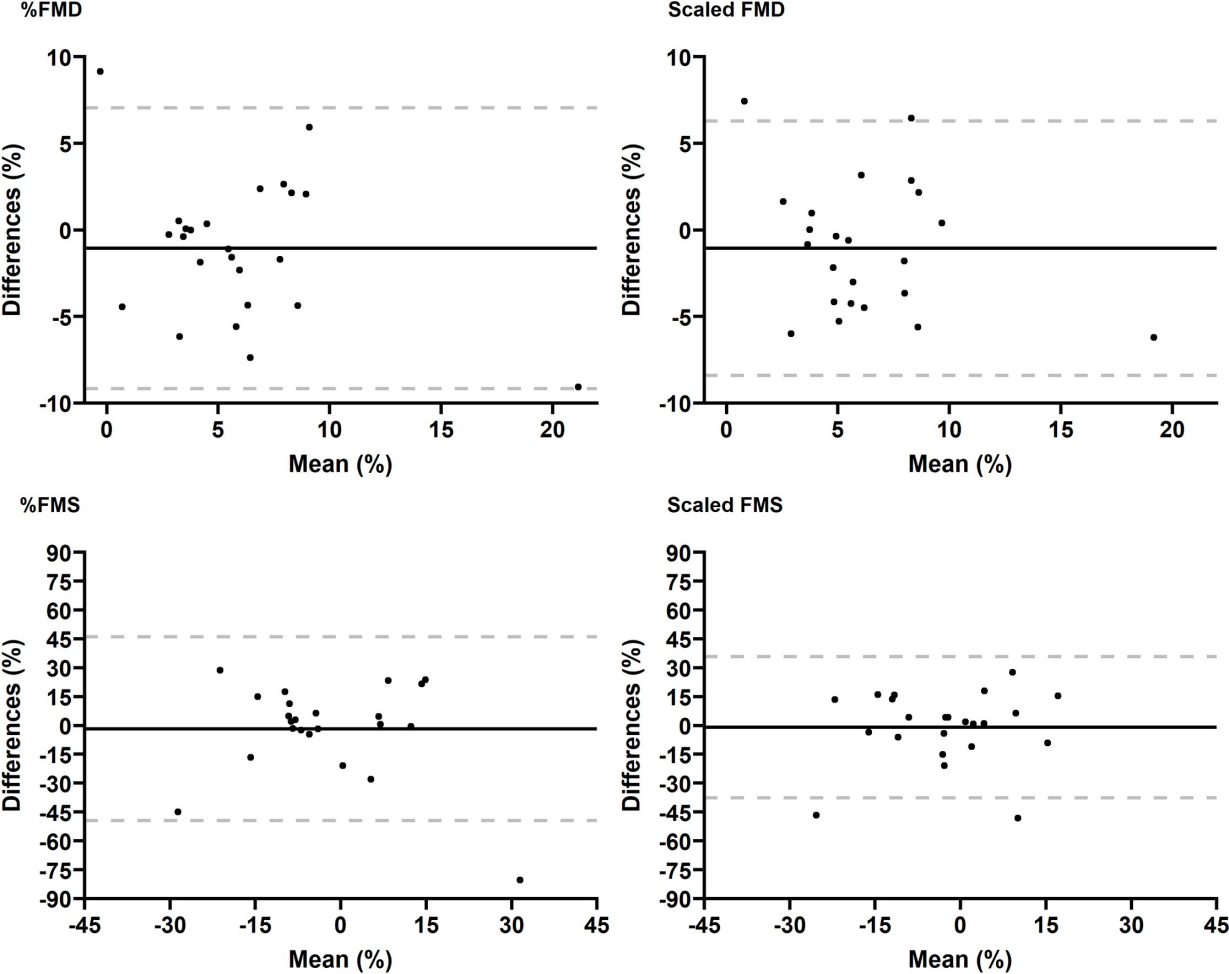
Bland-Altman plots for inter-day FMD and FMS measurements. Y-axis represents the difference between inter session measurements; the dashed grey lines correspond to the 95% LOA and the black line represent the mean of the differences; For %FMD, the lower 95% LOA was -9.16% (95% CI: - 12.18 to -6.14); and the upper 95% LOA was 7.04% (CI: 4.02 to 10.07); For scaled FMD, the lower 95% LOA was -8.39% (95% CI: -11.13 to -5.66); and the upper 95% LOA was 6.28% (95% CI: 3.54 to 9.01); For %FMS, the lower LOA was -49.50% (95% CI: -67.33 to -31.66); and the upper LOA was 46.08% (CI: 28.25 to 63.91); For scaled FMS, the lower LOA was -37.64% (CI: -51.32 to -23.96); and the upper LOA was 35.69% (95% CI: 22.01 to 49.38). FMS data were missing in 1 participant.

The Bland-Altman plots for inter-day FMS demonstrate poor repeatability, especially those of the 1^st^ and 2^nd^ min post-occlusion (S2 File). At the 3^rd^ min post occlusion, the bias of inter-day measurements were -1.70% (SD = 24.38) and -0.97% (SD = 18.71) for %FMS and scaled FMS, respectively. Bland-Altman plots for inter-day FMD showed a bias of -1.06% (SD = 4.13) and -1.06% (SD = 3.74), for %FMD and scaled FMD, respectively. Highpoints from visual plot inspection (Fig 2) include one participant that did not fall within the 95% LOA for %FMS, while two participants that fell outside these limits when FMS was scaled. Scaling FMS reduced the 95% LOA (- 37.64 to 35.69%) as compared to %FMS (- 49.50 to 46.07). One participant had missing data concerning inter-day FMS measures and was excluded from the final analysis of inter-day repeatability. Bland-Altman plots inter-day FMD showed no evidence of proportional bias and only one participant did not fall within the 95% LOA in %FMD and scaled FMD (Fig 2).

#### Association between FMD and FMS

No significant association was observed between %FMD and %FMS (r (20) = 0.04, *p* = 0.85) or scaled FMD and scaled FMS (r (20) = 0.03, *p* = 0.89). D_bas_ was moderately and negatively associated with %FMD (r (23) = - 0.38, *p* = 0.05), but not when FMD was scaled (r (23) = - 0.21, *p* = 0.30). Similarly, crPWV_bas_ showed a moderate negative association with %FMS (r (22) = - 0.56, *p* < 0.01), but not when FMS was scaled (r (20) = - 0.31, *p* = 0.15). crPWV_bas_ was not associated with *%*FMD (r (23) = - 0.13, *p* = 0.51), nor was there a significant association between D_bas_ and *%*FMS (r (20) = - 0.28, *p* = 0.20), or between D_bas_ and crPWV_bas_ (r (23) = 0.05, *p* = 0.82).

## Discussion

The main findings of this study were that both intra- and inter-day measurements of FMS, measured using carotid-radial arterial segments, demonstrated poor repeatability compared to FMD, which exhibited moderate-to-good repeatability. To our knowledge, this is the first study to assess the impact of allometric scaling on the repeatability of measurements of FMS and FMD. Scaling both FMS and FMD to crPWV and D_bas_, respectively, did not improve the overall repeatability of %FMS and %FMD.

Our findings contrast with those by Ellins et al. [16] who reported that %FMS had better inter-day repeatability compared to %FMD (CV: 7.3 vs 26.6%) in a group of 25 healthy adults aged 21–37 years. Multiple explanations might help clarify this disparity. First, we detected an erratic response of crPWV to repeated reactive hyperemia measures within participants, as denoted by decreases, increases, or no changes, in FMS. This explains the higher CV and the large 95% LOA. However, intra- and inter-day ICCs of repeated measurements of FMS are least moderate. This may be a function of ICC being prone to large sample variability rendering high correlations that might still represent an unacceptable measurement error [33, 34] (Atkinson and Nevill 1998; Hernaez 2016). Second, we hypothesize that FMS may be device-specific as the arterial segments, and the viscous-elastic properties of those arterial segments, and the algorithms used to determine time-transit differ between methods [21, 35, 36]. Furthermore, crPWV response to reactive hyperemia in the present study was quantified by piezoelectric pressure mechanotransducers, whereas Ellins et al. [16] used an oscillometric technique device (Vicorder) to measure brachial-radial PWV. Importantly, the viscous-elastic properties of these vascular beds may be different as peripheral arteries have less elastin content [37]. Thus, the piezoelectric pressure mechanotransducers technique may have missed decelerations in PWV from reactive hyperemia induce vasodilation of the brachial artery, compared to the oscillometric technique that measures PWV at a more local level. In addition, given that transit time is the main methodological confounder of PWV assessment, the differences in the distance measured between arterial sites in both methods likely yield different transit times and PWV estimates [21, 35, 36, 38].

crPWV responses to reactive hyperemia measured by applanation tonometry devices have demonstrated a consistent deceleration in PWV (negative FMS) [38–40], wherein the magnitude of deceleration was found to discriminate between participants with and without hypertension (normotensive: ~ -13.0% vs hypertensive: 1.1%) [39, 40]. We speculate that PWV response patterns to reactive hyperemia observed in the present study may be related to the cuff position used to induce supra-systolic brachial occlusion. The majority of the aforementioned studies used upper arm cuff-occlusion, whereas we used forearm cuff-occlusion, in line with the key studies on FMS [16, 17], and complying with the standardized FMD procedure [9]. To our knowledge, the influence of cuff-position on FMS has not yet been addressed. However, cuff-position in FMD induces different vasodilatory mechanisms [41], so we can only speculate that cuff position may also play a role in FMS response.

Although the decline in PWV is thought to be mediated by increased NO release [19, 20] and vessel diameter as suggested by the Moens–Korteweg equation, we found that %FMS was not associated with %FMD. This is in line with several studies evaluating FMS and artery diameters at rest [16, 17, 40, 42], but this is not an universal finding [43], and casts doubt on the currently accepted mechanism underpinning crPWV deceleration to reactive hyperemia [17].

Scaling improved intra-day but not inter-day variability of FMD. Thus, it seems that the influence of D_bas_ on FMD is more prominent in intra-day repeated measures. In addition, scaling eliminated the significant and negative association between D_bas_ and %FMD [6, 44]. This is important as %FMD is known to underestimate endothelial function in larger arteries likely due to a statistical artifact [14]. We also found a significant and moderate negative correlation between crPWV_bas_ and %FMS, and that %FMS was non-isometric (allometric coefficient < 0.6). However, scaling FMS did not improve intra and inter-day repeatability, although 95% LOA were smaller.

Our study is not without limitations. First, it is plausible that the use of multiple arterial segments (carotid, barchial radial) for the PWV measurements does not reflect the exact stiffness responses of the brachial artery to reactive hyperemia, given the poor agreement between local (e.g. carotid PWV) and regional measures of PWV (carotid-femoral PWV) [45]. In addition, the distance measurement between arterial sites of interest, required to regional PWV, is a main source of inaccuracy [21, 46], as the measurement of travel distances on the surface of the body may not accurately represent the true length and anatomy of the arterial segments. Future repeatibility studies should aim to resolve issue by using mathematical models derived from the Bramwell & Hill equation [47] to estimate PWV using a single arterial site [48] or local PWV of a single vascular bed [49]. Second, we did not measure crPWV continuously after cuff-deflation, which may have overlooked the true minimum value of crPWV in response to reactive hyperemia. However, it has been reported that crPWV response to reactive hyperemia remains significantly reduced during the first 180-s after cuff deflation [50]. Thus, it is unlikely that this contributed to the observed erratic crPWV response to reactive hyperemia and poor FMS reproducibility. Third, we did not evaluate endothelium-independent vasodilation in response to sublingual glyceryl trinitrate and consequently, we cannot ascertain the contribution of smooth muscle cells to brachial vasodilation and crPWV response to reactive hyperemia. Fourth, we did not include female participants and hence we cannot rule out the possibility of sex differences concerning FMD and FMS repeatability and different FMS response patterns, and our results cannot be generalized to both men and women. Future studies are needed to determine the repeatability of FMS in women. Finally, we did an à priori power analysis based on ICC results from highly specialized vascular laboratories, but this approach may not be adequate to other statistical measures of repeatability. In fact, according to Bland and Altman [51] the sample size for repeatability studies should be defined based on the amplitude of 95% CI of LOA. However, to our knowledge, none of the studies that assessed the repeatability of FMD and FMS has reported the 95% CI for the LOA.

## Conclusion

Our study demonstrated that FMS derived from piezoelectric pressure mechanotransducers placed in the carotid and radial arteries is not a repeatable method and showed poorer repeatability in comparison to brachial artery FMD as measured by echo-tracking, which exhibited moderate to good repeatability.

## Supporting information

S1 File Intra-day descriptive summary. (DOCX)

S2 File Bland Altman plots for FMS– 1^st^ and 2^nd^ minute following reactive hyperemia. (DOCX)

S3 File Reproducibility analysis of FMD and FMS stratified by age groups. (DOCX)

S4 File Inter-day descriptive summary. (DOCX)

S1 Data (SAV)
