# Supplementary material for: Flow-mediated slowing shows poor repeatability compared with flow-mediated dilation in non-invasive assessment of brachial artery endothelial function
Source: PLoS One. 2022 May 24;17(5):e0267287. doi: 10.1371/journal.pone.0267287 (PMC9129018; doi:10.1371/journal.pone.0267287)
Supplement: S1 File — (DOCX) [file pone.0267287.s001.docx]

| Supplement 1. Intra-day descriptive summary | | | | |  |  |
| --- | --- | --- | --- | --- | --- | --- |
| Variables | Mean | SD | Max | Min | IQR |  |
| *FMD (%)* |  |  |  |  |  |  |
| 1^st^ measure | 6.09 | 3.43 | 12.77 | 0.62 | 5.31 |  |
| 2^nd^ measure | 5.73 | 3.52 | 13.94 | -0.84 | 4.23 |  |
| *Scaled FMD (%)* |  |  |  |  |  |  |
| 1^st^ measure | 6.23 | 3.49 | 12.63 | - 0.12 | 5.38 |  |
| 2^nd^ measure | 5.63 | 3.49 | 13.27 | - 2.76 | 3.46 |  |
| *FMS_iii_ (%)* |  |  |  |  |  |  |
| 1^st^ measure | -1.23 | 10.25 | 28.57 | -14.41 | 8.53 |  |
| 2^nd^ measure | -1.97 | 12.29 | 6.67 | - 37.86 | 6.93 |  |
| *Scaled FMS_iii_ (%)* |  |  |  |  |  |  |
| 1^st^ measure | -1.23 | 10.04 | 28.09 | -11.62 | 11.35 |  |
| 2^nd^ measure | -6.45 | 10.36 | 9.36 | - 35.82 | 10.32 |  |
| *D_bas_ (mm)* |  |  |  |  |  |  |
| 1^st^ measure | 4.00 | 0.60 | 5.20 | 2.92 | 0.85 |  |
| 2^nd^ measure | 4.00 | 0.56 | 5.18 | 2.98 | 0.82 |  |
| *D_peak_ (mm)* |  |  |  |  |  |  |
| 1^st^ measure | 4.22 | 0.60 | 5.51 | 3.29 | 0.78 |  |
| 2^nd^ measure | 4.22 | 0.59 | 5.49 | 3.22 | 0.76 |  |
| *crPWV_bas_ (m/s)* |  |  |  |  |  |  |
| 1^st^ measure | 9.45 | 1.11 | 11.80 | 7.80 | 1.40 |  |
| 2^nd^ measure | 9.70 | 2.00 | 16.70 | 6.00 | 1.93 |  |
| *crPWV _iii_ (m/s)* |  |  |  |  |  |  |
| 1^st^ measure | 9.22 | 1.27 | 11.70 | 7.60 | 1.83 |  |
| 2^nd^ measure | 8.91 | 1.54 | 12.20 | 6.40 | 2.15 |  |
| *Abbreviations: SD: standard deviation; IQR: inter-quartile range; FMD: flow-mediated dilation; Dbas: brachial artery resting diameter; Dpeak: reactive hyperemia peak brachial artery diameter; FMSiii: flow-mediated slowing at 3rd-minute post-occlusion; crPWVbas: baseline carotid-radial pulse wave velocity; crPWViii: carotid-radial pulse wave velocity at 3rd-minute post-occlusion.* | | | | | | |
