# Supplementary material for: Flow-mediated slowing shows poor repeatability compared with flow-mediated dilation in non-invasive assessment of brachial artery endothelial function
Source: PLoS One. 2022 May 24;17(5):e0267287. doi: 10.1371/journal.pone.0267287 (PMC9129018; doi:10.1371/journal.pone.0267287)
Supplement: S2 File — (DOCX) [file pone.0267287.s002.docx]

**Supplement 2 – Bland Altman plots for FMS – 1^st^ and 2^nd^ minute following reactive hyperemia**

**
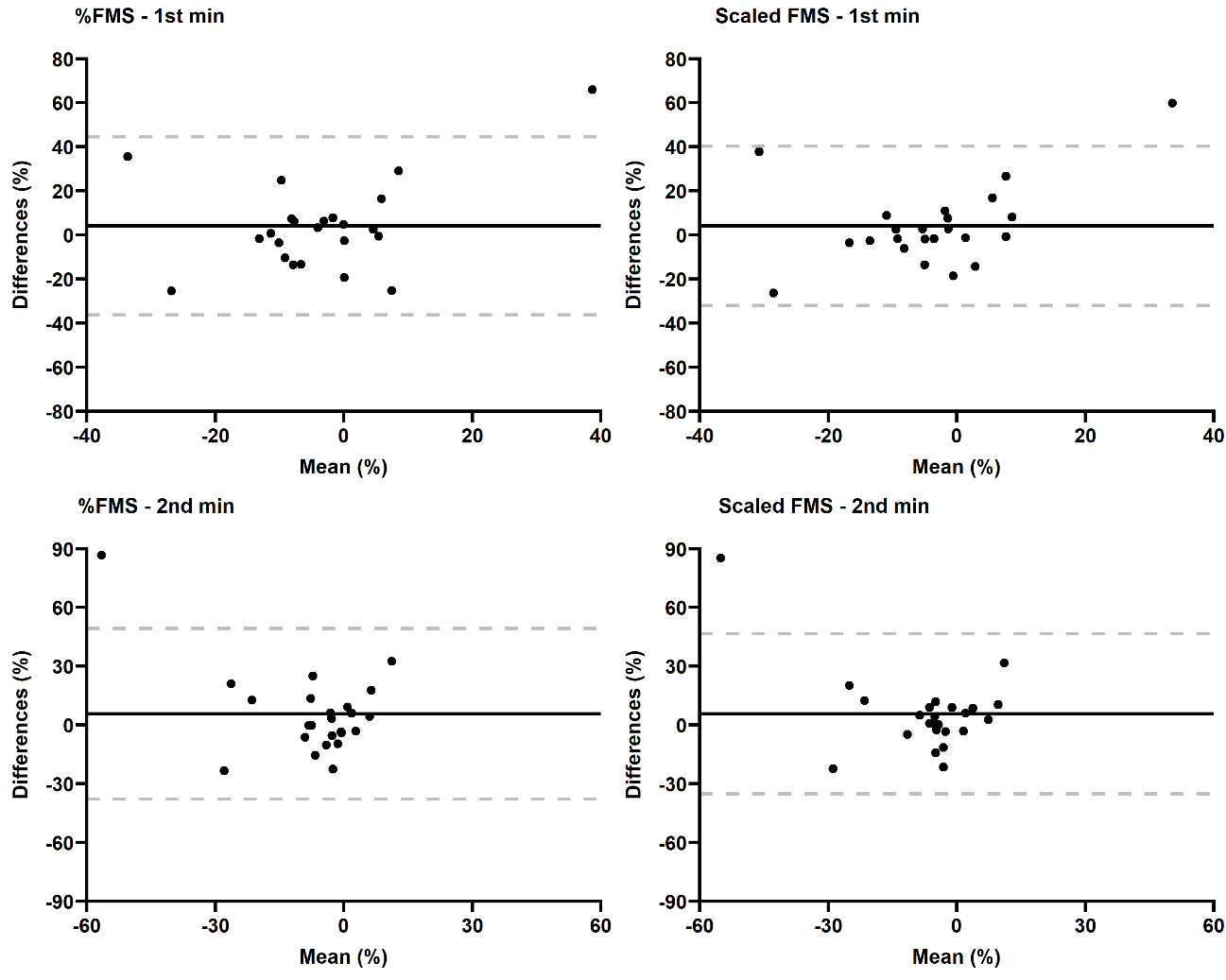
**

*Figure 3. Bland-Altman plots for intra-day %FMS and scaled FMS at first and second minute following reactive hyperemia. Y-axis represents the difference between the first and second measures; the dashed grey lines correspond to the 95% LOA and the black line represents the mean of the differences.*

*For %FMS at the first minute, the bias was 4.17% (95% CI -4.54 to 12.87), the lower LOA was -36.24 % (95% CI: -51.32 to -21.16); and the upper LOA was 44.60 % (CI 29.50 to 59.65). For scaled FMS at the first minute, the bias was 4.05% (95% CI: -3.74 to 11.83); the lower LOA was -32.10% (95% CI: -45.56 to -18.60); and the upper LOA was 40.20% (95% CI: 26.70 to 53.66). For %FMS at the second minute, the bias was 5.63% (95% CI: -3.75 to 15.01); the lower LOA was -37.93 % (95% CI: -54.20 to -21.67) and the upper LOA was 49.18% (95 % CI: 32.93 to 65.44). For the scaled FMS at the second minute, the bias was 5.63% (95% CI: -3.18 to 14.44); the lower LOA was -35.30% (95% CI: -50.54 to -20.01); and the upper LOA was 46.53 % (95% CI: 31.27 to 61.80). FMS data was missing in one participant.*


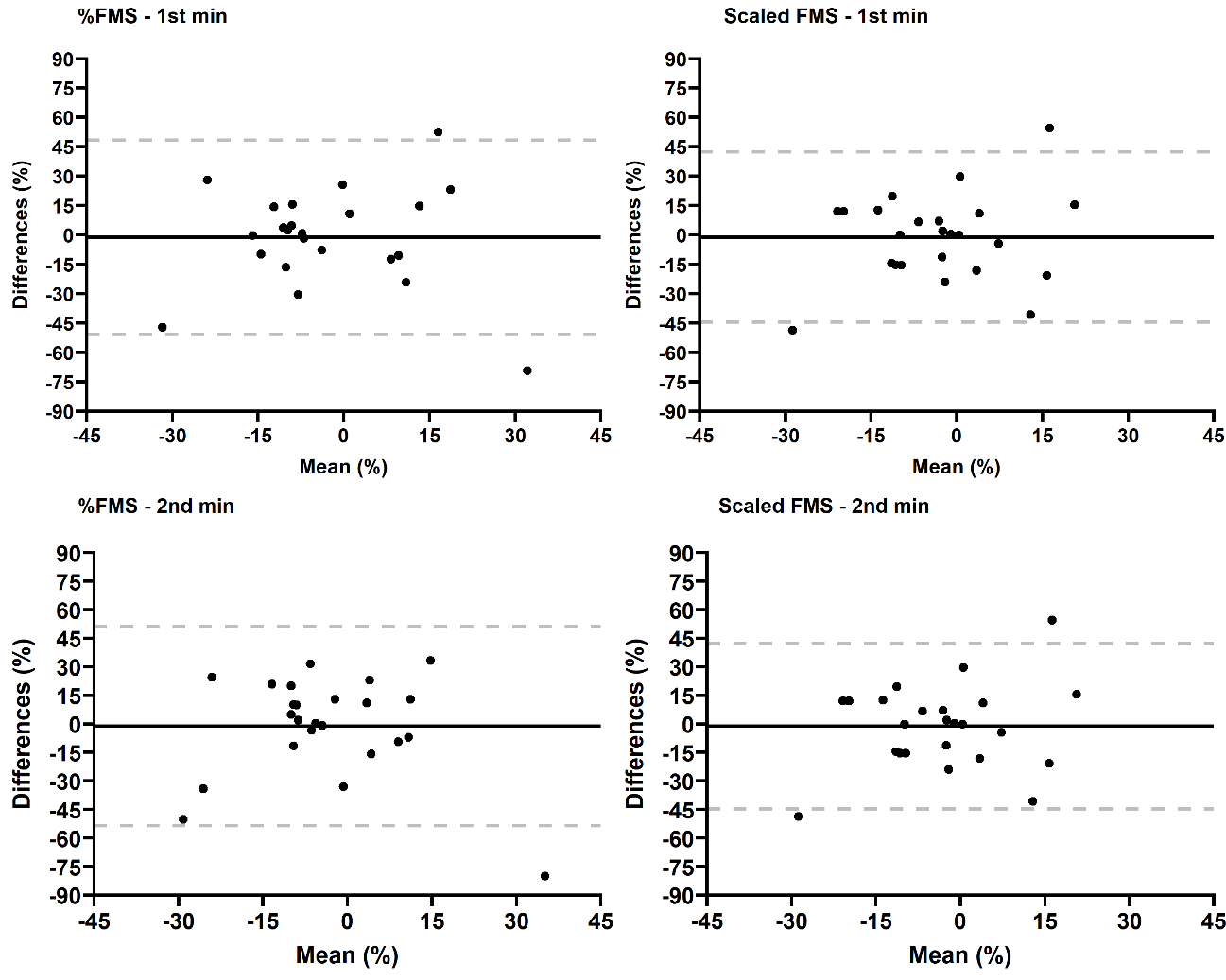


*Figure 4. Bland-Altman plots for inter-day %FMS and scaled FMS at first and second minute following reactive hyperemia. Y-axis represents the difference between the first and second measures; the dashed grey lines correspond to the 95% LOA and the black line represents the mean of the differences.*

*For %FMS at the first minute, the bias was -1.20% (95% CI -11.90 to 9.50), the lower LOA was -50.87% (95% CI: -69.40 to -32.34); and the upper LOA was 48.64 % (95%CI: 29.93 to 66.99). For scaled FMS at the first minute, the bias was -1.20% (95% CI: -10.56 to 8.16); the lower LOA was -32.10% (95% CI: -60.88 to -28.45); and the upper LOA was 40.20% (95% CI:26.04 to 58.47). For %FMS at the second minute, the bias was -1.09% (95% CI: -12.37 to 10.19); the lower LOA was -37.93 % (95% CI: -73.01 to -33.92) and the upper LOA was 49.18% (95 % CI: 31.74 to 70.83). For the scaled FMS at the second minute, the bias was -1.09% (95% CI: -9.67 to 7.49); the lower LOA was -35.30% (95% CI: -55.78 to -26.06); and the upper LOA was 46.53 % (95% CI:23.8 to 69.48). FMS data was missing in one participant.*
