# Supplementary material for: Flow-mediated slowing shows poor repeatability compared with flow-mediated dilation in non-invasive assessment of brachial artery endothelial function
Source: PLoS One. 2022 May 24;17(5):e0267287. doi: 10.1371/journal.pone.0267287 (PMC9129018; doi:10.1371/journal.pone.0267287)
Supplement: S3 File — (DOCX) [file pone.0267287.s003.docx]

**Supplement 3.** Reproducibility analysis of FMD and FMS stratified by age groups

| Table 1. Intra-day reproducibility | | | | | |
| --- | --- | --- | --- | --- | --- |
|  | | | | 95% ICC Confidence Interval | |
| Variables | CV (%) | ICC | Lower bound | | Upper bound |
| *FMD (%)* | *18* | *0.83* | *0.60* | | *0.93* |
| Young | 15 | 0.85 | 0.44 | | 0.96 |
| Older | 20 | 0.82 | 0.59 | | 0.92 |
| *Scaled FMD (%)* | *13* | *0.83* | *0.60* | | *0.93* |
| Young | 13 | 0.81 | 0.33 | | 0.95 |
| Older | 13 | 0.84 | 0.22 | | 0.93 |
| Absolute FMD (mm) | 20 | 0.80 | 0.73 | | 0.90 |
| Young | 14 | 0.81 | 0.33 | | 0.95 |
| Older | 25 | 0.80 | 0.42 | | 0.97 |
| *FMS_iii_ (%)* | *136* | *0.82* | *0.56* | | *0.93* |
| Young | 118 | 0.29 | -2.45 | | 0.84 |
| Older | 149 | 0.55 | -0.36 | | 0.87 |
| *Scaled FMS_iii_ (%)* | *180* | *0.55* | *-0.05* | | *0.80* |
| Young | 111 | 0.48 | -0.90 | | 0.85 |
| Older | 255 | 0.66 | -0.18 | | 0.94 |
| *D_bas_ (mm)* | *3* | *0.99* | *0.97* | | *0.99* |
| Young | 2 | 0.98 | 0.92 | | 0.99 |
| Older | 3 | 0.99 | 0.95 | | 0.99 |
| *D_peak_ (mm)* | *3* | *0.97* | *0.94* | | *0.99* |
| Young | 3 | 0.94 | 0.80 | | 0.98 |
| Older | 2 | 0.99 | 0.97 | | 0.99 |
| *crPWV_bas_ (m/s)* | *7* | *0.65* | *0.19* | | *0.85* |
| Young | 9 | 0.64 | -0.34 | | 0.90 |
| Older | 6 | 0.75 | 0.18 | | 0.93 |
| *crPWV _iii_ (m/s)* | *8* | *0.76* | *0.43* | | *0.90* |
| Young | 7 | 0.66 | -0.34 | | 0.91 |
| Older | 9 | 0.82 | 0.33 | | 0.95 |

Intraclass correlation coefficients (ICC) with 95% confidence intervals and coefficients of variation (CV) were calculated over two measurements. Abbreviations: FMD: flow-mediated dilation; FMSiii: flow-mediated slowing at 3rd-minute post-occlusion; D_bas_: brachial artery resting diameter; Dpeak: reactive hyperemia peak brachial artery diameter; crPWV: carotid-radial pulse wave velocity at 3rd-minute post-occlusion. Older adults >60 years; young adults: 18 to 30 years

| Table 2. Inter-day reproducibility stratified by age group | | | | |
| --- | --- | --- | --- | --- |
|  |  |  | 95% ICC Confidence Interval | |
| Variables | CV (%) | ICC | 95% CI Lower bound | 95% CI Upper bound |
| *FMD (%)* | *25* | *0.78* | *0.48* | *0.90* |
| Young | 38 | 0.73 | 0.02 | 0.92 |
| Older | 16 | 0.82 | 0.34 | 0.95 |
| *Scaled FMD (%)* | *31* | *0.80* | *0.55* | *0.91* |
| Young | 40 | 0.78 | 0.19 | 0.94 |
| Older | 25 | 0.81 | 0.31 | 0.95 |
| *FMS_iii_ (%)* | *145* | *0.18* | *-1.03* | *0.66* |
| Young | 165 | -0.05 | -3.77 | 0.53 |
| Older | 123 | 0.38 | -1.63 | 0.84 |
| *Scaled FMS_iii_ (%)* | *145* | *0.32* | *-0.66* | *0.72* |
| Young | 138 | 0.20 | -2.40 | 0.78 |
| Older | 152 | 0.44 | -1.35 | 0.85 |
| *D_bas_ (mm)* | *3* | *0.96* | *0.92* | *0.98* |
| Young | 3 | 0.93 | 0.77 | 0.98 |
| Older | 3 | 0.97 | 0.87 | 0.99 |
| *D_peak_ (mm)* | *3* | *0.96* | *0.91* | *0.98* |
| Young | 3 | 0.95 | 0.84 | 0.99 |
| Older | 3 | 0.97 | 0.85 | 0.99 |
| *crPWV_bas_ (m/s)* | *8* | *0.58* | *0.07* | *0.82* |
| Young | 10 | 0.47 | - 0.97 | 0.85 |
| Older | 7 | 0.32 | -1.30 | 0.80 |
| *crPWV _iii_ (m/s)* | *11* | *0.46* | *-0.26* | *0.77* |
| Young | 10 | 0.47 | -0.97 | 0.87 |
| Older | 12 | 0.48 | -0.94 | 0.86 |

Intraclass correlation coefficients (ICC) with 95% confidence intervals and coefficients of variation (CV) were calculated over two measurements. Abbreviations: FMD: flow-mediated dilation; FMSiii: flow-mediated slowing at 3rd-minute post-occlusion; D_bas_: brachial artery resting diameter; Dpeak: reactive hyperemia peak brachial artery diameter; crPWV: carotid-radial pulse wave velocity at 3rd-minute post-occlusion. Older adults >60 years; young adults: 18 to 30 years
