# Supplementary material for: Flow-mediated slowing shows poor repeatability compared with flow-mediated dilation in non-invasive assessment of brachial artery endothelial function
Source: PLoS One. 2022 May 24;17(5):e0267287. doi: 10.1371/journal.pone.0267287 (PMC9129018; doi:10.1371/journal.pone.0267287)
Supplement: S4 File — (DOCX) [file pone.0267287.s004.docx]

| Supplement 4. Inter-day descriptive summary | | | | | |
| --- | --- | --- | --- | --- | --- |
| Variables | Mean | SD | Max | Min | IQR |
| *FMD (%)* |  |  |  |  |  |
| 1^st^ measurement | 5.45 | 3.90 | 16.62 | -1.53 | 3.96 |
| 2^nd^ measurement | 6.50 | 5.17 | 25.69 | -4.86 | 4.32 |
| *Scaled FMD (%)* |  |  |  |  |  |
| 1^st^ measurement | 5.45 | 3.87 | 16.08 | -2.21 | 3.78 |
| 2^nd^ measurement | 6.50 | 4.65 | 22.29 | -2.92 | 3.53 |
| *FMS_iii_ (%)* |  |  |  |  |  |
| 1^st^ measurement | -3.18 | 16.13 | 26.74 | -51.06 | 11.55 |
| 2^nd^ measurement | -2.27 | 19.85 | 71.67 | -35.54 | 13.94 |
| *Scaled FMS_iii_ (%)* |  |  |  |  |  |
| 1^st^ measurement | -3.11 | 15.34 | 24.77 | -48.68 | 15.63 |
| 2^nd^ measurement | -2.27 | 13.67 | 34.08 | -28.8 | 13.63 |
| D_bas_ *(mm)* |  |  |  |  |  |
| 1^st^ measurement | 4.14 | 0.55 | 5.35 | 3.32 | 0.88 |
| 2^nd^ measurement | 4.08 | 0.60 | 5.34 | 3.18 | 0.74 |
| D_peak_ *(mm)* |  |  |  |  |  |
| 1^st^ measurement | 4.36 | 0.58 | 5.61 | 3.29 | 0.85 |
| 2^nd^ measurement | 4.33 | 0.57 | 5.58 | 3.35 | 0.64 |
| *crPWV_bas_ (m/s)* |  |  |  |  |  |
| 1^st^ measurement | 8.96 | 1.09 | 10.80 | 6.80 | 1.75 |
| 2^nd^ measurement | 9.33 | 1.28 | 12.10 | 6.00 | 1.53 |
| *crPWV _iii_ (m/s)* |  |  |  |  |  |
| 1^st^  measurement | 8.64 | 1.52 | 11.4 | 4.6 | 1.70 |
| 2^nd^ measurement | 8.94 | 1.05 | 11.20 | 6.9 | 1.50 |
| *Abbreviations: SD: standard deviation; IQR: inter-quartile range; FMD: flow-mediated dilation; D_bas_: brachial artery resting diameter; D_peak_: reactive hyperemia peak brachial artery diameter; FMS_iii_: flow-mediated slowing at 3rd-minute post-occlusion; crPWV_bas_: baseline carotid-radial pulse wave velocity; crPWV_iii_: carotid-radial pulse wave velocity at 3rd-minute post-occlusion.* | | | | | |
